# Hyperviscosity Syndrome as a Rare Presentation of Immunoglobulin G Kappa Smoldering Multiple Myeloma Successfully Treated With Daratumumab

**DOI:** 10.14740/jmc5398

**Published:** 2026-08-25

**Authors:** Muralidhar Idamakanti, Rani Indrani Bijjam, Alexei Bakhirev, Manoj Kumar

**Affiliations:** a Adult Internal Medicine Services (AIMS), Presbyterian Healthcare Services (PHS), Albuquerque, NM 87106, USA; b Pathology Associates of Albuquerque (PAA), Albuquerque, NM 87106, USA; c Department of Hematology/Oncology, Presbyterian Healthcare Services (PHS), Albuquerque, NM 87106, USA

**Keywords:** Hyperviscosity syndrome, Smoldering multiple myeloma, Waldenström macroglobulinemia, paraproteinemia, hematuria, retinal hemorrhage, epistaxis, Daratumumab

## Abstract

Hyperviscosity syndrome (HVS) is an oncologic emergency most commonly associated with Waldenstrom macroglobulinemia and immunoglobulin (Ig)M paraproteinemia. Clinically, significant HVS in smoldering multiple myeloma (SMM), particularly IgG-associated disease without overt CRAB (hypercalcemia, renal insufficiency, anemia, and bone lesions) criteria, is exceedingly rare. We report a unique case of IgG kappa smoldering myeloma complicated by symptomatic HVS, manifesting with retinal hemorrhage, epistaxis, and gross hematuria, despite the absence of overt end-organ myeloma-defining events. A 70-year-old patient with Sjogren syndrome and longstanding monoclonal gammopathy showed gradual progression from high-risk monoclonal gammopathy of undetermined significance (MGUS) to smoldering myeloma over several years. Bone marrow biopsy showed 10% plasma cells, with no high-risk cytogenetic abnormalities on limited fluorescence *in situ* hybridization (FISH) testing. Positron emission tomography/computed tomography (PET/CT) imaging repeatedly showed no fluorodeoxyglucose (FDG)-avid osseous disease. In the setting of HVS, serum viscosity peaked at 5.7 centipoise, with concomitant IgG elevation to 5,463 mg/dL and an M-protein of 3.01 g/dL. Given symptomatic hyperviscosity, treatment with daratumumab was initiated despite the absence of SLiM (≥ 60% clonal plasma cells, light chain ratio ≥ 100, and magnetic resonance imaging (MRI) focal lesions)-CRAB criteria for overt multiple myeloma. Following therapy, serum viscosity rapidly improved from 5.7 to 2.2, with corresponding reductions in IgG and M-protein levels and resolution of bleeding manifestations. This case highlights that symptomatic hyperviscosity may occur in IgG smoldering myeloma at a relatively modest plasma cell burden and may itself represent a clinically meaningful indication for early therapeutic intervention.

## Introduction

Hyperviscosity syndrome (HVS) is a hematologic emergency caused by increased resistance to blood flow from elevated serum proteins or cellular components [1]. The syndrome classically presents with a triad of mucosal bleeding, visual abnormalities, and neurologic manifestations due to impaired microcirculatory perfusion [2]. Common clinical manifestations include epistaxis, retinal hemorrhage, blurred vision, headache, dizziness, ataxia, and altered mental status [3].

HVS is most commonly associated with Waldenstrom macroglobulinemia, owing to the intravascular distribution and pentameric structure of immunoglobulin (Ig)M paraproteins, which substantially increase serum viscosity even at relatively low concentrations [4]. In contrast, hyperviscosity associated with IgG monoclonal gammopathies is considerably less common because IgG molecules are smaller and more widely distributed into the extravascular compartment [5, 6].

Smoldering multiple myeloma (SMM) is an intermediate asymptomatic plasma cell neoplasm characterized by serum monoclonal protein ≥ 3 g/dL and/or 10–60% clonal bone marrow plasma cells in the absence of myeloma-defining SLiM-CRAB (≥ 60% clonal bone marrow plasma cells, light chain ratio ≥ 100, MRI focal lesions, hypercalcemia, renal insufficiency, anemia, and bone lesions) criteria [7]. Although patients with high-risk SMM carry a substantial risk of progression to symptomatic multiple myeloma, they typically lack end-organ manifestations at diagnosis [8]. HVS occurring in SMM is exceedingly rare, particularly in IgG-associated disease without extensive marrow involvement or overt myeloma-defining organ damage [9].

Recent advances in risk stratification and early intervention strategies for high-risk SMM have led to increasing interest in plasma cell-directed therapy before progression to overt multiple myeloma [10]. Daratumumab, an anti-CD38 monoclonal antibody, has demonstrated promising efficacy in delaying disease progression in high-risk SMM, including in the phase III AQUILA trial, which evaluated daratumumab versus active monitoring in high-risk smoldering multiple myeloma [11].

We report a rare case of IgG kappa smoldering myeloma complicated by clinically significant HVS, manifesting as retinal hemorrhage, epistaxis, and gross hematuria despite absence of overt CRAB criteria. The patient demonstrated marked improvement in serum viscosity and clinical manifestations following initiation of daratumumab therapy.

## Case Report

This is a 70-year-old male with a history of Sjogren syndrome, who was initially evaluated in June 2018 for elevated globulin levels and found to have an IgG kappa monoclonal gammopathy, with an M-protein of 1.18 g/dL and a free light chain ratio of 3.39. Over subsequent years, serial laboratory monitoring demonstrated a gradual progression of paraproteinemia, with rising M-protein levels and worsening free light chain ratio (Tables 1, 2).

**Table 1:** Chronologic Disease Progression

Date	Findings
June 2018	IgG kappa paraprotein 1.18 g/dL; FLC ratio 3.39
August 2020	Bone marrow biopsy: 6% plasma cells
2020–2025	Progressive increase in M-protein and FLC ratio
June 2025	Bone marrow biopsy: 10% plasma cells; rouleaux formation
January 2026	Gross hematuria requiring bladder irrigation
February 2026	Serum viscosity 5.7 centipoise
March 2026	Retinal hemorrhage and epistaxis
April 2026	Daratumumab initiated
June 2026	Serum viscosity improved to 2.2

IgG: immunoglobulin G; FLC: free light chain.

**Table 2:** Selected Hematologic and Myeloma Laboratory Data

Date	WBC (× 10^3^/µL)	Hemoglobin (g/dL)	Platelets (× 10^3^/µL)	IgG (mg/dL)	M-protein (g/dL)
August 2020	3.2	13.8	121	—	2.21
August 2023	3.3	12.6	148	—	2.51
April 2024	3.5	13.3	120	—	2.68
June 2025	—	—	—	5,463	3.01
January 2026	2.5	12.2	105	—	—
April 2026	3.0	12.7	114	—	—
May 2026	3.1	13.7	118	—	2.26
June 22026	5.4	15.2	141	2,653	1.35

WBC: white blood cell; IgG: immunoglobulin G.

**Figure 1:**
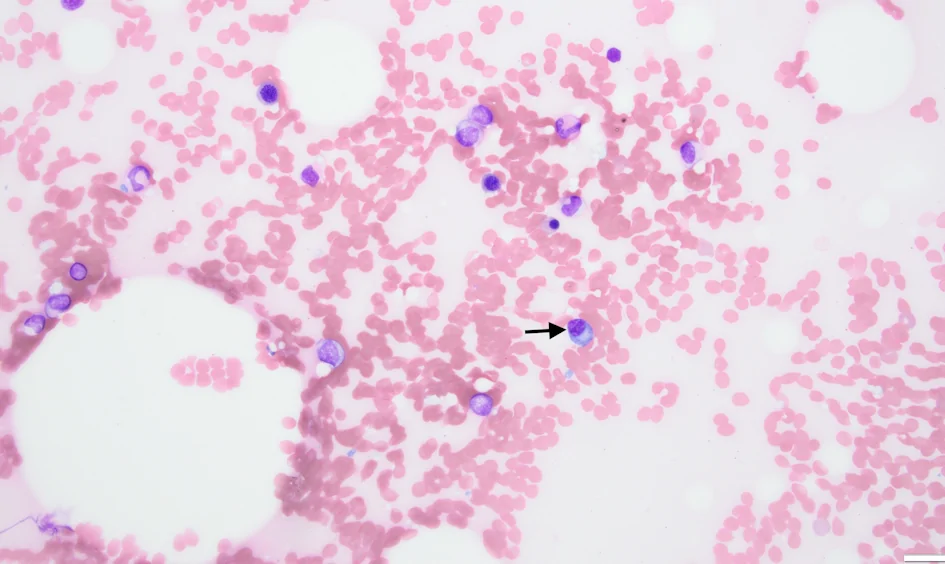
Bone marrow biopsy (with Wright-Giemsa staining) at initial diagnosis of high-risk MGUS demonstrating approximately 6% plasma cells (black arrow), consistent with high-risk MGUS. MGUS: monoclonal gammopathy of undetermined significance.

**Figure 2:**
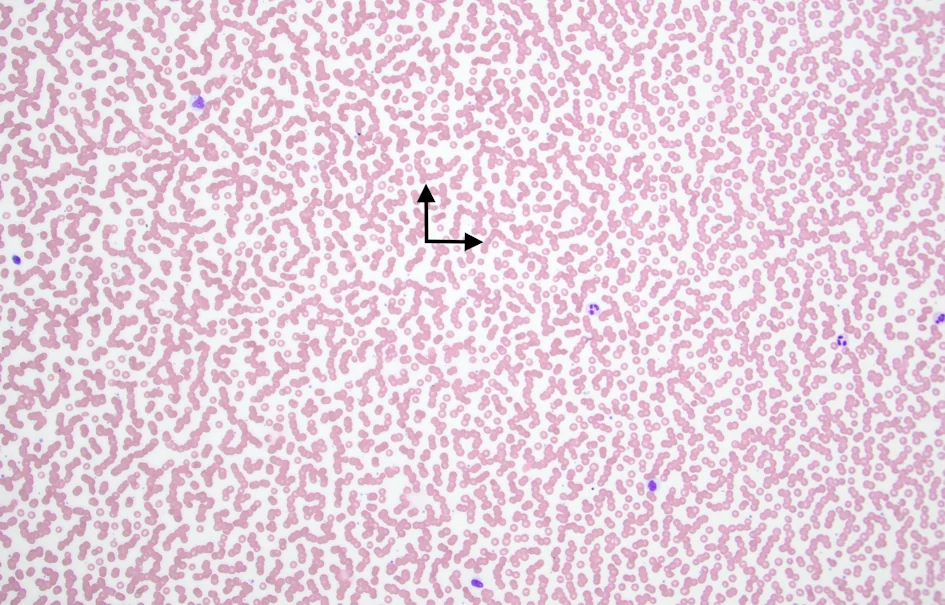
Peripheral blood smear (with Wright-Giemsa staining) demonstrating well-formed rouleaux (Black arrows).

Bone marrow biopsy performed in August 2020 demonstrated 6% plasma cells, consistent with high-risk monoclonal gammopathy of undetermined significance (MGUS) (Fig. 1). Peripheral blood smear showed rouleaux formation without any evidence of agglutination (Fig. 2). Skeletal imaging, including radiographs and serial positron emission tomography/computed tomography (PET/CT) studies, repeatedly showed no lytic lesions or fluorodeoxyglucose (FDG)-avid osseous disease.

**Figure 3:**
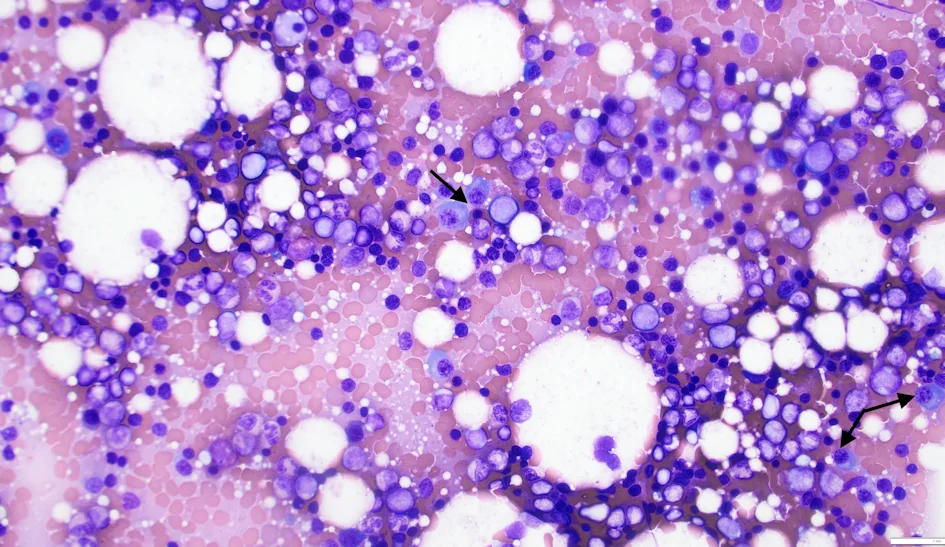
Bone marrow biopsy (with Wright-Giemsa staining) showing approximately 10% plasma cells (black arrows), meeting diagnostic criteria, and demonstrating progression to smoldering multiple myeloma.

**Figure 4:**
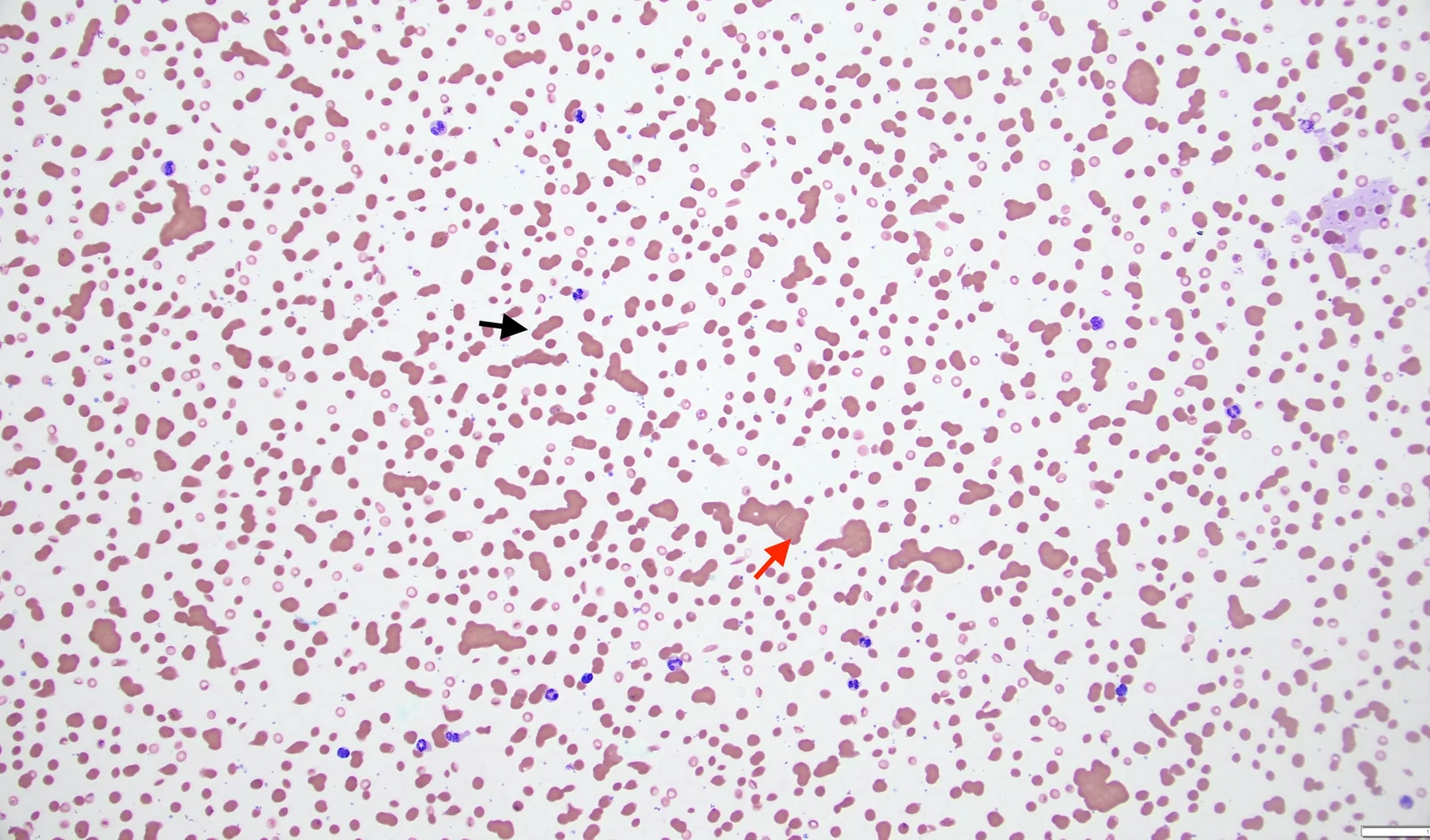
Peripheral blood smear (with Wright-Giemsa staining) demonstrating rouleaux formation (black arrows) and red blood cell agglutination (red arrow) associated with elevated monoclonal immunoglobulin levels and hyperviscosity syndrome.

By June 2025, a repeat bone marrow biopsy demonstrated progression to SMM, with approximately 10% plasma cells and 2% monoclonal kappa-restricted plasma cells by flow cytometry. The cells expressed CD138, CD38, and CD56 (Fig. 3). The peripheral blood smear showed rouleaux formation and red blood cell agglutination, suggesting a significant rheologic disturbance associated with paraproteinemia (Fig. 4). Fluorescence *in situ* hybridization (FISH) studies were negative for tumor protein p53 (TP53) deletion, 1q gain, and 1p loss, though additional testing was limited by insufficient specimen.

Despite the absence of classic CRAB criteria, the patient developed progressive signs suggestive of HVS. In January 2026, the patient presented to the emergency department with gross hematuria and lower abdominal discomfort, necessitating Foley catheter placement and continuous bladder irrigation. Imaging revealed prostatomegaly and bladder wall thickening without evidence of urinary tract malignancy or obstructive pathology. Coagulation studies, including prothrombin time (PT), partial thromboplastin time (PTT), fibrinogen, and factor X activity, were within normal limits. Subsequently, the patient developed retinal hemorrhage and recurrent epistaxis. Further evaluations in February 2026 revealed markedly elevated serum viscosity of 5.7 centipoise. Laboratory evaluation showed an IgG elevation to 5,463 mg/dL, with M-protein at 3.01 g/dL and total protein at 10.9 g/dL (Tables 1–3). PET/CT imaging remained negative for active myelomatous lesions.

**Table 3:** Serum Viscosity Trend Following Daratumumab Therapy

Date	Serum viscosity (cP)
February 18, 2026	5.7
Treatment initiated on April 8, 2026	
April 20, 2026	4.5
April 27, 2026	3.7
May 4, 2026	3.4
May 18, 2026	2.5
June 1, 2026	2.2

cP: centipoise.

**Figure 5:**
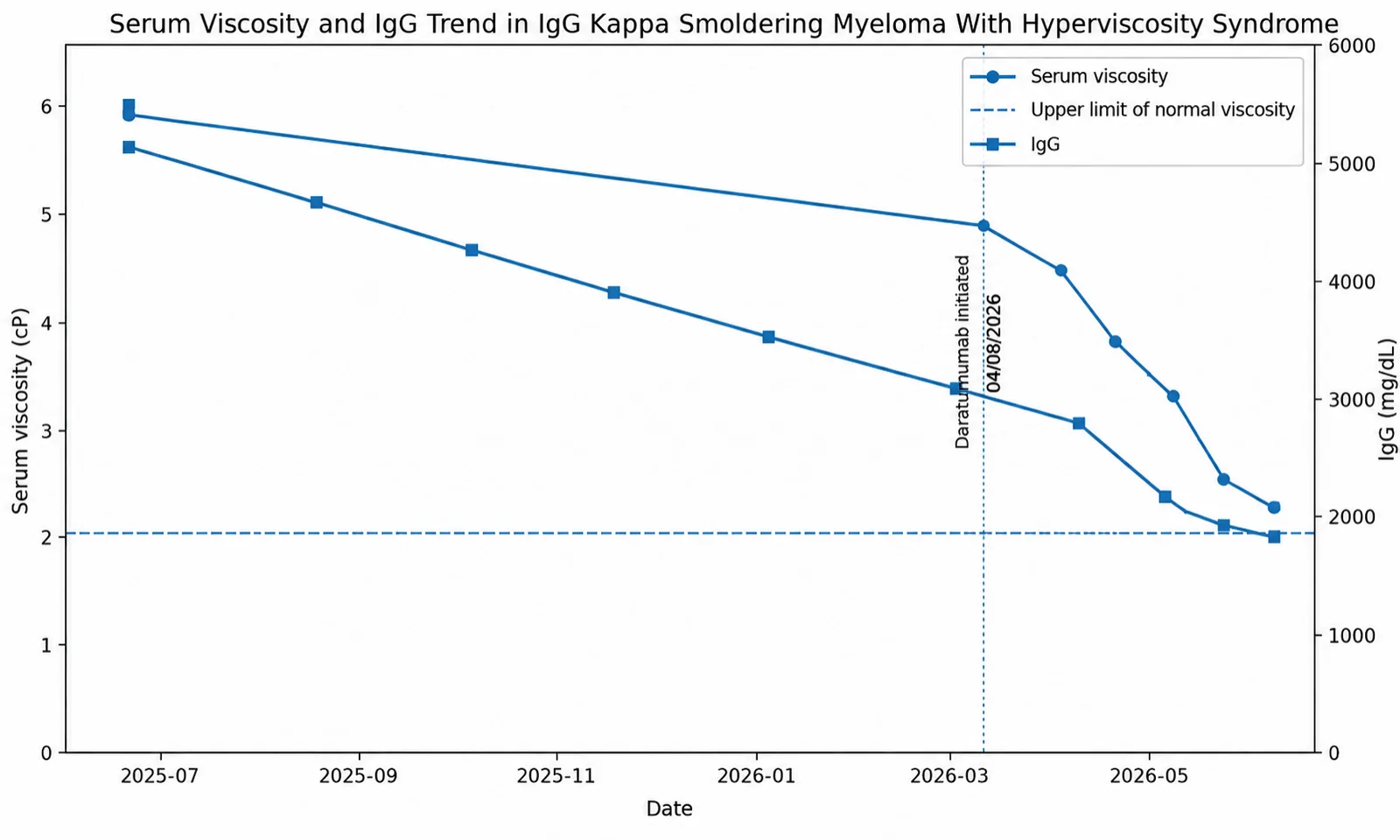
Trends in serum viscosity and IgG levels following initiation of daratumumab therapy. Trends consistent with a significant hematologic response and resolution of hyperviscosity-related symptoms. Ig:

Given symptomatic hyperviscosity attributed to progressive smoldering myeloma, treatment was recommended despite the absence of overt multiple myeloma-defining events to prevent recurrence of HVS and related complications. The patient had previously declined therapy and pursued a holistic approach but agreed to initiate daratumumab in April 2026 after extensive counseling. After treatment initiation, the patient experienced rapid biochemical improvement. Serum viscosity decreased from 5.7 to 2.2 over approximately 3 months, accompanied by reductions in IgG from 5,463 mg/dL to 2,653 mg/dL and in M-protein from 3.01 g/dL to 1.35 g/dL (Table 3, Fig. 5). Cytopenias improved significantly, with no recurrence of bleeding or HVS episodes and no treatment-limiting toxicities reported (Tables 1, 3). These findings were confirmed by repeat eye and physical examinations.

## Discussion

HVS is an uncommon but potentially life-threatening complication of plasma cell dyscrasias characterized by impaired blood flow due to increased serum viscosity from circulating paraproteins [1]. The classic clinical triad includes mucosal bleeding, visual disturbances, and neurologic manifestations [2]. HVS is most frequently associated with Waldenstrom macroglobulinemia because IgM is a large pentameric immunoglobulin predominantly confined to the intravascular compartment [4]. Common causes of HVS are included in Table 4. In contrast, symptomatic HVS secondary to IgG paraproteinemia is rare and generally requires substantially higher immunoglobulin concentrations [5].

**Table 4:** Common Causes of Hyperviscosity Syndrome

Underlying disorder	Predominant paraprotein/cause	Frequency of HVS	Typical serum viscosity threshold	Comments
Waldenstrom macroglobulinemia	IgM monoclonal protein	Most common cause	Often > 4 cP (may occur at lower levels)	IgM is pentameric and remains largely intravascular, making it highly viscosity-inducing. Approximately 10–30% of patients develop symptomatic HVS during the disease course.
Multiple myeloma (IgA)	IgA monoclonal protein	Common among myeloma-associated HVS	Usually > 4–5 cP	IgA tends to form dimers and polymers, increasing serum viscosity despite lower concentrations than IgG.
Multiple myeloma (IgG)	IgG monoclonal protein	Uncommon	Usually > 5–6 cP	Most cases occur with very high IgG concentrations. IgG3 has the greatest tendency toward self-aggregation and hyperviscosity.
PCL	High circulating plasma cell burden with IgG or IgA paraproteinemia	Uncommon but well recognized	Variable	Hyperviscosity results from both markedly elevated monoclonal protein levels and circulating plasma cells. Patients often present with aggressive disease and extensive extramedullary involvement.
Smoldering multiple myeloma	Usually IgG or IgA	Extremely rare	Variable	Symptomatic HVS without SLiM-CRAB criteria is exceptional. The present case represents this rare presentation.
MGUS	Low-level monoclonal protein	Very rare	Rarely clinically significant	Usually insufficient paraprotein burden to produce symptomatic HVS.
Cryoglobulinemia	Cryoglobulins	Uncommon	Not serum viscosity dependent	Increased viscosity occurs primarily at low temperatures due to protein precipitation rather than absolute paraprotein concentration.
Polycythemia vera	Increased erythrocyte mass	Uncommon	Hematocrit > 60–65%	Hyperviscosity results from increased cellular mass rather than paraproteins.
Leukostasis (AML/CML)	Extreme leukocytosis	Rare	WBC typically > 100–300 × 10^9^/L	Symptoms result from impaired microvascular flow caused by circulating leukemic blasts rather than serum protein abnormalities.

HVS: hyperviscosity syndrome; IgG: immunoglobulin G; IgA: immunoglobulin A; IgM: immunoglobulin M; PCL: plasma cell leukemia; MGUS: monoclonal gammopathy of undetermined significance; AML: acute myeloid leukemia; CML: chronic myeloid leukemia; WBC: white blood cell; cP: centipoise; SLiM-CRAB: ≥ 60% clonal bone marrow plasma cells, light chain ratio ≥ 100, magnetic resonance imaging focal lesions, hypercalcemia, renal insufficiency, anemia, and bone lesions.

SMM is defined by the presence of serum monoclonal protein ≥ 3 g/dL and/or 10–60% clonal plasma cells in the bone marrow in the absence of myeloma-defining SLiM-CRAB features [7] (Table 5). While SMM is associated with risk of progression to overt multiple myeloma, clinically significant HVS occurring before transformation is exceedingly uncommon [8].

**Table 5:** Myeloma-Defining CRAB Criteria

CRAB feature	Definition (IMWG criteria)	Clinical significance
C: hypercalcemia	Serum calcium > 11 mg/dL or > 1 mg/dL above the upper limit of normal	Indicates increased osteoclastic bone resorption due to active myeloma
R: renal insufficiency	Serum creatinine > 2 mg/dL or estimated creatinine clearance < 40 mL/min	Reflects myeloma-related kidney injury, often from light chain nephropathy
A: anemia	Hemoglobin < 10 g/dL or > 2 g/dL below the lower limit of normal	Results from bone marrow infiltration and impaired erythropoiesis
B: bone lesions	One or more osteolytic lesions identified on skeletal survey, CT, or PET/CT	Represents myeloma-related bone destruction and skeletal involvement

CRAB: hypercalcemia, renal insufficiency, anemia, and bone lesions; IMWG: International Myeloma Working Group; CT: computed tomography; PET/CT: positron emission tomography/computed tomography.

Our patient developed multiple manifestations consistent with symptomatic HVS, including retinal hemorrhage, recurrent epistaxis, and gross hematuria, in the setting of elevated serum viscosity of 5.7 centipoise. Importantly, alternative causes of bleeding were not identified. Coagulation parameters, including PT, PTT, fibrinogen, and factor X activity, were within normal limits, and extensive urologic imaging failed to demonstrate structural pathology accounting for recurrent hematuria.

Ocular manifestations are among the most characteristic findings of HVS and are often considered highly suggestive of clinically significant disease [12]. Retinal venous engorgement, flame hemorrhages, cotton wool spots, and papilledema may result from impaired retinal microcirculation [13, 14]. The retinal hemorrhage observed in this patient strongly supported clinically significant hyperviscosity rather than an isolated laboratory abnormality.

An additional notable feature of this case is the relatively modest marrow plasma cell burden despite severe rheologic complications. Bone marrow biopsy demonstrated approximately 10% plasma cells, barely meeting diagnostic criteria for smoldering myeloma, yet the patient developed marked symptomatic hyperviscosity. This finding suggests that factors beyond tumor burden alone, including immunoglobulin subtype, polymerization tendencies, molecular interactions, and intravascular distribution, may substantially influence serum viscosity [5].

Peripheral blood smear findings showed rouleaux formation and red blood cell agglutination, both classic hematologic manifestations of significant paraproteinemia [15, 16]. Rouleaux formation occurs when monoclonal proteins reduce the zeta potential between erythrocytes, promoting aggregation and impairing microvascular flow [15, 16].

Management should be individualized based on symptom severity, progression risk, comorbidities, and treatment goals. Urgent plasmapheresis is traditionally considered first-line therapy for acute, severe HVS. However, plasmapheresis provides only a rapid, temporary reduction in paraprotein levels; durable control requires therapy directed at the underlying plasma-cell clone [11, 17]. The AQUILA trial demonstrated improved progression-free survival with daratumumab monotherapy in patients with high-risk smoldering myeloma [11]. In high-risk smoldering myeloma, alternatives to daratumumab include lenalidomide alone or lenalidomide plus dexamethasone, both of which have shown delayed progression in randomized trials. More intensive regimens based on proteasome inhibitors or multidrug combinations remain investigational and may carry greater toxicity [17].

However, symptomatic hyperviscosity in smoldering myeloma remains poorly described in the literature and may be an underrecognized indication for early therapeutic intervention. Daratumumab, a monoclonal antibody targeting CD38, has demonstrated substantial efficacy in plasma cell dyscrasias through direct cytotoxicity, immune modulation, and depletion of clonal plasma cells [18]. Daratumumab monotherapy was selected in this case for its anti-plasma-cell activity, favorable tolerability, and evidence supporting early treatment in high-risk smoldering myeloma [17]. After initiating daratumumab, our patient experienced rapid improvement in serum viscosity from 5.7 to 2.2 centipoise, with corresponding reductions in IgG and M-protein levels. Importantly, retinal hemorrhage, epistaxis, and hematuria resolved with therapy, strongly supporting a causal relationship between paraproteinemia and hyperviscosity in this case.

Cases of HVS associated with IgG smoldering myeloma remain exceedingly rare in the literature. Most reported cases of hyperviscosity in plasma cell disorders involve overt multiple myeloma, particularly IgM/IgA-associated disease, due to increased polymerization [6]. This case therefore underscores the importance of maintaining clinical suspicion for HVS even in non-IgM plasma cell disorders without overt CRAB features or extensive marrow involvement. It also demonstrates that symptomatic hyperviscosity may warrant early therapeutic intervention in otherwise asymptomatic smoldering myeloma, and that timely initiation of plasma cell-directed therapy may prevent irreversible ophthalmologic and/or neurologic complications.

### Conclusions

This case describes a rare presentation of IgG kappa SMM complicated by clinically significant HVS, manifesting as retinal hemorrhage, epistaxis, and gross hematuria. Despite the absence of overt CRAB features and a relatively low marrow plasma cell burden, the patient developed severe, symptomatic hyperviscosity, with serum viscosity reaching 5.7 centipoise. Daratumumab therapy led to rapid improvement in serum viscosity and clinical symptoms. This case highlights the importance of recognizing HVS in IgG-associated intermediate plasma cell disorders and suggests that symptomatic hyperviscosity may warrant treatment initiation in selected patients.

### Learning points

**Figure 6:**
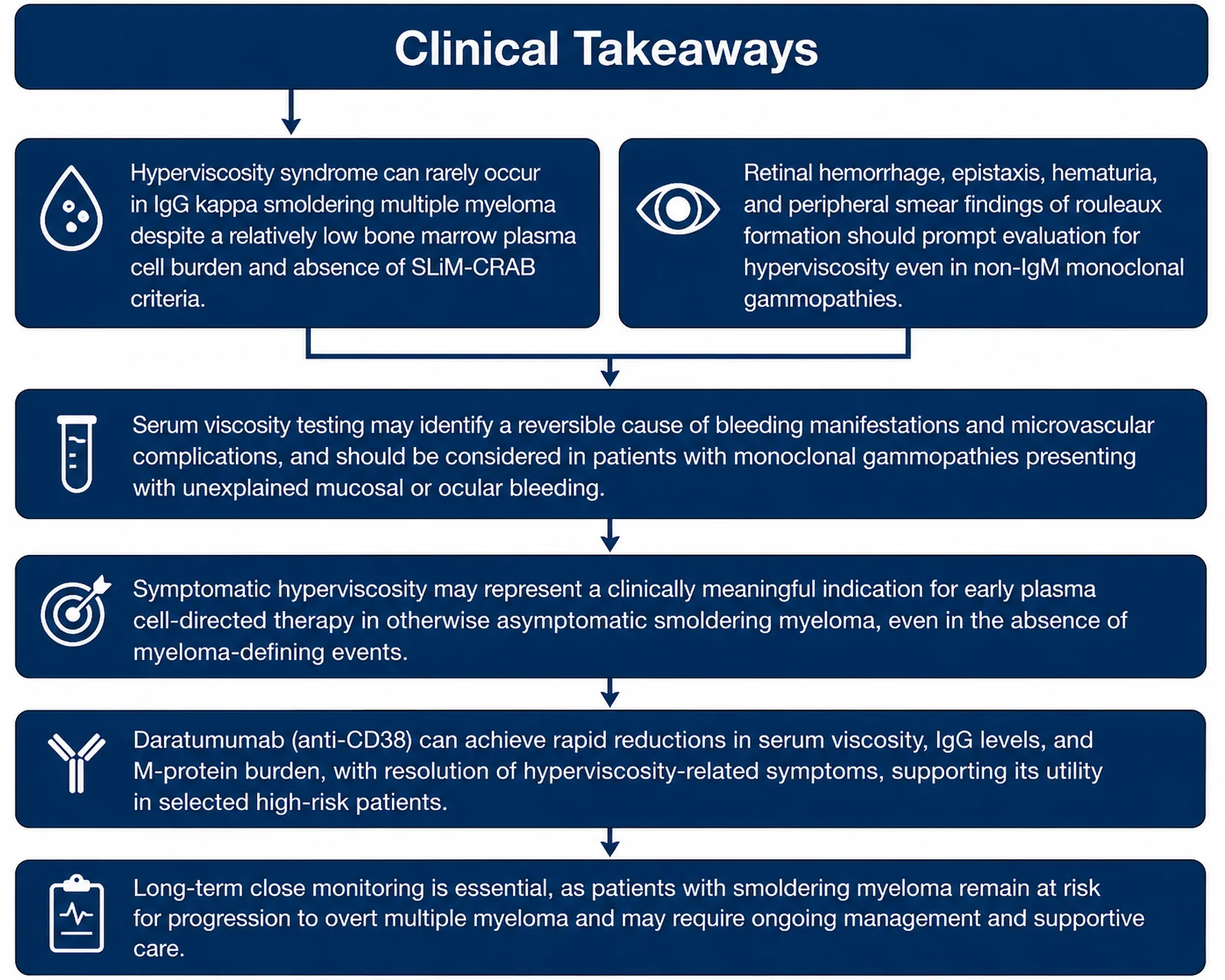
Learning points.

Clinically significant HVS can occur in IgG kappa SMM despite the absence of SLiM-CRAB criteria. Retinal hemorrhage, epistaxis, hematuria, or rouleaux formation should prompt evaluation for hyperviscosity. This case highlights symptomatic hyperviscosity as a potential indication for early plasma cell-directed therapy, with daratumumab producing rapid clinical and serologic improvement (Fig. 6).

## Data Availability

The authors declare that the data supporting the findings of this case report are available within the article. All data analyzed during this study were obtained from previously published studies and are available in the public domain.

## Abbreviations

HVS: hyperviscosity syndrome
SMM: smoldering multiple myeloma
MGUS: monoclonal gammopathy of undetermined significance
IgG: immunoglobulin G
IgM: immunoglobulin M
CRAB: hypercalcemia, renal insufficiency, anemia, and bone lesions
SLiM: ≥ 60% clonal plasma cells, light chain ratio ≥ 100, and magnetic resonance imaging focal lesions
PET/CT: positron emission tomography/computed tomography
FDG: fluorodeoxyglucose
FISH: fluorescence *in situ* hybridization
TP53: tumor protein p53
CD: cluster of differentiation
PT: prothrombin time
PTT: partial thromboplastin time
WBC: white blood cell
cP: centipoise
IMWG: International Myeloma Working Group
anti-CD38: anti-cluster of differentiation 38 monoclonal antibody
